# Pathogenesis of chronic migraine: the role of neuromodulators

**DOI:** 10.1186/1129-2377-16-S1-A38

**Published:** 2015-09-28

**Authors:** Giovanni D'Andrea

**Affiliations:** Research & Innovation, Padua, Italy

The pathogenesis of chronic migraine (CM) remains largely unknown. We hypothesized that anomalies of tyrosine metabolism, found in migraine without aura (MwoA) patients, play an important role in the transformation of MwoA into CM, since the increase in the number of MwoA attacks is the most predisposing factor for the occurrence of CM. To test our hypothesis we measured the plasma levels of dopamine (DA), noradrenaline (NE) and trace amines, including tyramine (TYR) and octopamine (OCT), in a group of 73 patients with CM, 13 patients with chronic tension-type headache (CTTH) and 37 controls followed in the Headache Centres of the Neurology Departments of Asti, Milan and Vicenza hospitals in Italy.

The plasma levels of DA and NE were several-fold higher in CM patients compared with control subjects (p > 0.001). The plasma levels of TYR were also extremely elevated (p > 0.001); furthermore, these levels progressively increased with the duration of the CM. Our data support the hypothesis that altered tyrosine metabolism plays an important role in the pathogenesis of CM. The high plasma levels of TYR, a potent agonist of the trace amine associated receptors type 1 (TAAR1), may ultimately down-regulate this receptor because of loss of inhibitory presynaptic regulation, therein resulting in uncontrolled neurotransmitter release. This may produce functional metabolic consequences in the synaptic clefts of the painmatrix implicated in CM.

Written informed consent to publication was obtained from the patient(s).

